# Evidence of SARS-CoV-2-Specific Memory B Cells Six Months After Vaccination With the BNT162b2 mRNA Vaccine

**DOI:** 10.3389/fimmu.2021.740708

**Published:** 2021-09-28

**Authors:** Annalisa Ciabattini, Gabiria Pastore, Fabio Fiorino, Jacopo Polvere, Simone Lucchesi, Elena Pettini, Stefano Auddino, Ilaria Rancan, Miriam Durante, Michele Miscia, Barbara Rossetti, Massimiliano Fabbiani, Francesca Montagnani, Donata Medaglini

**Affiliations:** ^1^ Laboratory of Molecular Microbiology and Biotechnology, Department of Medical Biotechnologies, University of Siena, Siena, Italy; ^2^ Department of Medical Biotechnologies, University of Siena, Siena, Italy; ^3^ Department of Medical Sciences, Infectious and Tropical Diseases Unit, University Hospital of Siena, Siena, Italy

**Keywords:** vaccination, SARS-CoV-2, COVID-19, BNT162b2 vaccine, memory B cells

## Abstract

SARS-CoV-2 mRNA vaccines have demonstrated high efficacy and immunogenicity, but limited information is currently available on memory B cell generation and long-term persistence. Here, we investigated spike-specific memory B cells and humoral responses in 145 subjects, up to 6 months after the BNT162b2 vaccine (Comirnaty) administration. Spike-specific antibodies peaked 7 days after the second dose and significant antibody titers and ACE2/RBD binding inhibiting activity were still observed after 6 months, despite a progressive decline over time. Concomitant to antibody reduction, spike-specific memory B cells, mostly IgG class-switched, increased in the blood of vaccinees and persisted 6 months after vaccination. Following the *in vitro* restimulation, circulating memory B cells reactivated and produced spike-specific antibodies. A high frequency of spike-specific IgG^+^ plasmablasts, identified by computational analysis 7 days after boost, positively correlated with the generation of IgG^+^ memory B cells at 6 months. These data demonstrate that mRNA BNT162b2 vaccine elicits strong B cell immunity with spike-specific memory B cells that still persist 6 months after vaccination, playing a crucial role for a rapid response to SARS-CoV-2 virus encounter.

## Introduction

Severe acute respiratory syndrome coronavirus 2 (SARS-CoV-2), the agent responsible for Coronavirus Disease 2019 (COVID-19), has infected more than 280 million individuals and is currently responsible for almost 4.7 million of deaths (https://covid19.who.int/). An unprecedented effort has been made in the development of effective vaccines, essential to prevent further morbidity and mortality. In total, 17 different vaccines are currently used worldwide following the authorization by national regulatory authorities, 7 of which have received the approval for emergency use by the WHO regulatory authority (https://extranet.who.int/pqweb/sites/default/files/documents/Status_COVID_VAX_19August2021.pdf). As of September 20, 2021, a total of 5,776,127,976 vaccine doses have been administered worldwide.

Two SARS-CoV-2 vaccines based on the novel messenger RNA (mRNA) technology, BNT162b2 (produced by Pfizer-BioNTech, commercially named Comirnaty) and mRNA-1273 (produced by Moderna), have been licensed for human use. This is the first time that mRNA vaccines have been approved for human use, and limited information is available on the profile and persistence of the elicited immune response. Phase 3 trials of these vaccines have shown an efficacy of 94–95% at preventing symptomatic infection after two doses administered 3–4 weeks apart (1, 2). Two recent studies of BNT162b2 vaccine effectiveness against SARS-CoV-2 infection and COVID-19 cases, in a nationwide mass vaccination setting, demonstrate that the vaccine is effective for a wide range of COVID-19 related outcomes, such as hospitalization, severe illness, and death (3, 4) confirming data of effectiveness reported in the randomized phase III clinical study (NCT04368728) (1).

However, available data on the immune response elicited by mRNA vaccination are mostly related to antibody responses (5–7) while limited information is available on the persistence of memory B cells that are expected to play a crucial role for a rapid response to SARS-CoV-2 infection. The human memory B-cell compartment is indeed a pillar for vaccine efficacy, since it is the basis for protective immunity upon pathogen encounter leading to the generation of plasma cells capable to produce spike specific antibodies. Therefore, the assessment of memory B cells provides a critical biomarker to profile the long-term persistence of effective immune responses even beyond the decline of antibody titers.

How long memory B cell responses elicited by mRNA COVID-19 vaccines will persist still remains, therefore, a critical open question. To answer this question, we have investigated the generation and persistence of peripheral spike-specific memory B cells and circulating antibodies up to 6 months after vaccination with the BNT162b2 vaccine in subjects naïve to the SARS-CoV-2 infection.

## Material and Methods

### Study Design

Plasma and peripheral blood mononuclear cells (PBMCs) samples were obtained from 145 healthcare workers (HCWs) aged 24–75 years who received two doses of the BNT162b2 (Pfizer-BioNTech; Comirnaty) vaccine 3 weeks apart, as reported in **Table S1**. Exclusion criteria included pregnancy, previous documented SARS-CoV-2 infection, and immunocompromising comorbidities (congenital, acquired, or drug-related). All participants provided a written informed consent before participation to the study. Study participants were recruited at the Infectious and Tropical Diseases Unit, Azienda Ospedalier0 Universitaria Senese (Siena, Italy). The study was performed in compliance with all relevant ethical regulations and the protocol was approved by the local Ethical Committee for Clinical experimentation of Regione Toscana Area Vasta Sud Est (CEASVE), protocol code 18869 IMMUNO_COV v1.0 of November 18, 2020, approved on the December 21, 2020. Clinical data collection and management were carried out using the software REDCap (Research Electronic Data Capture, Vanderbilt University).

### Plasma and Peripheral Blood Mononuclear Cells Isolation

Venous blood samples were collected in heparin-coated blood tubes (BD Vacutainer) at the baseline (day 0), at days 7, 21 (pre-boost), 28 (7 days post-boost), 90 (3 months after the first vaccination dose), and 160–180 (5–6 months after the first vaccination dose). PBMCs were isolated by density-gradient sedimentation, using Ficoll-Paque (Lymphoprep, Voden Medical Instrument, Meda, Italy). Isolated PBMC were then cryopreserved in a cell recovery medium [10% DMSO (Thermo Fisher Scientific) and 90% heat inactivated fetal bovine serum (Sigma Aldrich)] and stored in liquid nitrogen until used. Plasma samples were stored at -80°C.

### Enzyme-Linked Immunosorbent Assay

Maxisorp microtiter plates (Nunc, Denmark) were coated with recombinant wild type or mutated (lineage B.1.1.7, Alpha; B.1.351/B.1.351.2/B.1.351.3, Beta; P.1/P.1.1/P.1.2, Gamma; B.1.617, Delta) SARS-CoV-2 full spike protein (S1+S2 ECD), or spike RBD (all from Sino Biological) with 50 μl per well of 1 μg/ml protein solution in PBS (Sigma-Aldrich) overnight at 4°C. Plates were blocked at room temperature (RT, 20–25°C) for 1 h with 200 μl of 5% skimmed milk powder, 0.05% Tween 20, 1 × PBS. All plasma samples, heated at 56°C for 1 h to reduce the risk from any potential residual virus, were added and titrated in two-fold dilutions in duplicate in a diluent buffer (3% skimmed milk powder, 0.05% Tween 20, 1 × PBS for 1 h at RT. Anti-human horseradish peroxidase (HRP)-conjugated antibodies for IgG (diluted 1:6,000), IgM, IgA (diluted 1:4,000), IgG1, IgG2, IgG3, IgG4 (diluted 1:2,000; all from Southern Biotechnology) were added in a diluent buffer for 1 h at RT. Plates were developed with 3,3’,5,5’-Tetramethylbenzidine (TMB; Thermo Fisher Scientific) substrate for 10 min at RT, followed by the addition of 1 M stop solution. Absorbance at 450 nm was measured on Multiskan FC Microplate Photometer (Thermo Fisher Scientific). A WHO international positive control (plasma from a vaccinated donor diluted 1:5,000; NIBSC) and negative control (plasma from an unvaccinated donor diluted 1:20, NIBSC) were added in duplicate to each plate as the internal control for assay reproducibility. Antibody end point titers were expressed as the reciprocal of the sample dilution reporting double the background OD value.

### ACE2/RBD Inhibition Assay

ACE2/RBD inhibition was tested with a SARS-CoV-2 surrogate virus neutralization test (sVNT) kit (cPass™, Genscript), according to the protocol of the manufacturer. Briefly, plasma samples, positive and negative controls were diluted 1:10 in a dilution buffer, mixed 1:1 with the HRP-RBD buffer, and incubated for 30 min at 37°C. Then, 100 µl of each mixture were added to each well of ACE2-coated flat-bottom 96-well plates and incubated for 15 min at 37°C. Plates were washed four times with a wash solution and tapped dry. A total of 100 µl of TMB solution was added to each well and plates were developed for 15 min at RT. After that, the reaction was quenched by adding the stop solution (50 µl to each well) and the absorbance at 450 nm was measured on the Multiskan FC Microplate Photometer (Thermo Fisher Scientific). Results of the ACE2/RBD inhibition assay are expressed as follows: percentage inhibition = (1−sample OD value/negative control OD value) * 100. Inhibition values ≥30% are regarded as positive results, while values <30% as negative results, as indicated by the manufacturer.

### Multiparametric Flow Cytometry

Two million of PBMCs were incubated with the BD human Fc block (BD Biosciences) for 10 min at RT. Cells were stained with the SARS-CoV-2 spike full protein ECD-His recombinant biotinylated-protein (25 µg/ml, Sino Biological) in the staining Buffer [PBS, 0.5% Bovine Serum Albumin (BSA) and 2 mM EDTA, all from Sigma-Aldrich] for 30 min at 4°C, and then stained with FITC-conjugated streptavidin for 30 min at 4°C. Cells were washed and stained for 30 min at 4°C with the following antibody cocktail, containing: CD3-PECy 7 (clone SK7), CD56-PECy7 (clone B159), CD14-PECy7 (clone M5E2), CD19-BUV395 (clone SJ25C1), IgM-BV421 (clone G20-127), IgD-PE (clone IA6-2), CD11c-BB700 (clone 3.9), CXCR5-BV650 (clone RF8B2), CD27-APC-R700 (clone M-T271), CD24-BV786 (clone ML5), CD38-BUV737 (clone HB7), IgG-BV711 (clone G18-145), (all from Becton Dickinson), and IgA-APC (clone IS11-8E10, Miltenyi Biotec). Following surface staining, cells were washed once with PBS and labeled with Live/Dead FSV780 according to the instructions of the manufacturer (BD Biosciences). Cells were washed with PBS, and incubated at 4°C for 15 min in the dark in 100 µl of BD fixation solution (BD Biosciences). All antibodies were titrated for an optimal dilution. About 1–2 × 10^6^ cells were acquired and stored for each sample with the SO LSRFortessa X20 flow cytometer (BD Biosciences). Data analysis was performed using FlowJo v10 (TreeStar, USA).

### Memory B Cell ELISpot

PBMCs were collected from HCWs 160–180 days following the first dose of mRNA BTN162b2 vaccination and evaluated for IgM and IgG production using the Human IgM/IgG Double-Color Enzymatic ELISpot assay (CTL Europe GmbH, Bonn, Germany). The protocol was performed according to the instructions of the manufacturer. PBMCs were cultured in a complete RPMI medium at a concentration of 2 × 10^6^ PBMCs/ml in 24-well tissue culture plates and stimulated with polyclonal B cell stimulator (B-Poly-S, diluted 1:1,000) in the CTL-Test B medium for 4 days, at 37°C with 5% CO_2_ to induce antibody production from resting memory B cells (MBC). After stimulation, cells were harvested and counted using the automated cell counter (Bio-Rad Laboratories, USA). Multiscreen filter 96-well plates were pre-wetted with 70% ethanol and then coated with the recombinant wild type SARS-CoV-2 spike full protein ECD (Sino Biological, 10 μg/ml) for the detection of antigen-specific IgG and IgM or with anti-Ig capture antibody for the detection of total IgG and IgM overnight at 4°C. Coating with an unrelated antigen was included. Coated wells were washed with PBS, and 50 µl of the CTL-Test B medium supplemented with 1% L-glutamine (Sigma Aldrich) were added to each well. In a volume of 50 µl/well of CTL-Test B medium, 1 × 10^6^, 2 × 10^5^, 4 × 10^4^ and 1 × 10^5^, 2 × 10^4^, 4 × 10^3^ pre-stimulated cells were seeded to evaluate the spike-specific and total Ig, respectively. After incubation at 37°C in the presence of 5% CO_2_, cells were removed by washing with PBS-0.05% Tween 20, and anti-human IgM/IgG detection solution was added for 3 h at RT. Plates were washed, incubated with 80 µl/well of Tertiary Solution (FITC-HRP and Strep-AP, both diluted 1:1,000) for 1 hr at RT, washed again, and Blue and Red Developer Solutions were added, each for 15 min. The reaction was stopped by an extensive washing in tap water, and plates were dried in the dark at RT. The number of spots was determined by plate scanning and analysis performed with an Immunospot S6 Ultimate Analyzer (CTL Europe GmbH).

### Computational Flow Cytometry Analysis

The B cell population analyzed in our data set was gated as live, singlet, CD3^−^/CD14^-^/CD56^-^ CD19^+^ spike^+^ cells using FlowJo v10 (TreeStar, USA). The analysis was then carried on the R platform (v4.0.3). Flow cytometry standard (FCS) files were exported as uncompensated data in the R environment as a flowSet object (list of FCS), that was then compensated with the FlowCore package 2.0.1 (8) and logicle transformed (9) using the estimateLogicle function for automatic parameters selection for each fluorescence marker. Clustering analysis was performed following the FlowSOM function pipeline (FlowSOM package v1.20.0). Marker expression was normalized with z-score and grid size was set to 7 x 7. Similar nodes were merged in 10 metaclusters (metaclustering step). The Euclidean distance was used in both the FlowSOM clustering and metaclustering. Thresholds to bisect positive and negative cells for each marker expression were automatically set with the flowDensity package (10). FlowSOM results were displayed as a heatmap reporting the percentage of positive cells for each marker within the metacluster as previously described (11).

### Statistics

Kruskal-Wallis test, followed by Dunn’s post-test for multiple comparisons, was used to assess the statistical differences of ELISA titers and ACE2/RBD inhibition percentages at different time points post vaccination. Pearson test was used to evaluate the correlation between log-transformed ELISA titers and ACE2/RBD inhibition for each time point. Kruskal-Wallis test, followed by Dunn’s post-test for multiple comparisons, was used for assessing the statistical difference between the frequencies of Spike-specific B cells assessed by the flow cytometry data. Mann–Whitney test was used for assessing the statistical difference between the clusters at different time points on the data of computational flow cytometry analysis. *P*-values were corrected for multiple tests with the Benjamini-Hochberg False Discovery Rate (FDR) method (12). Statistical significance was defined as FDR < 10^-2^. Multiple correlations between metaclusters were performed using Spearman’s correlation with the psych package and visualized with the corrplot package; p-values were corrected with the Benjamini-Hockberg FDR method. Mann–Whitney test, followed by Dunn’s post-test for multiple comparisons, was used for assessing the statistical difference between Spike-specific and unrelated antigen-specific B cells in the ELISPOT data. A P-value ≤ 0.05 was considered significant. Analyses were performed using GraphPad Prism v9 (GraphPad Software, USA).

## Results

**Figure 1:**
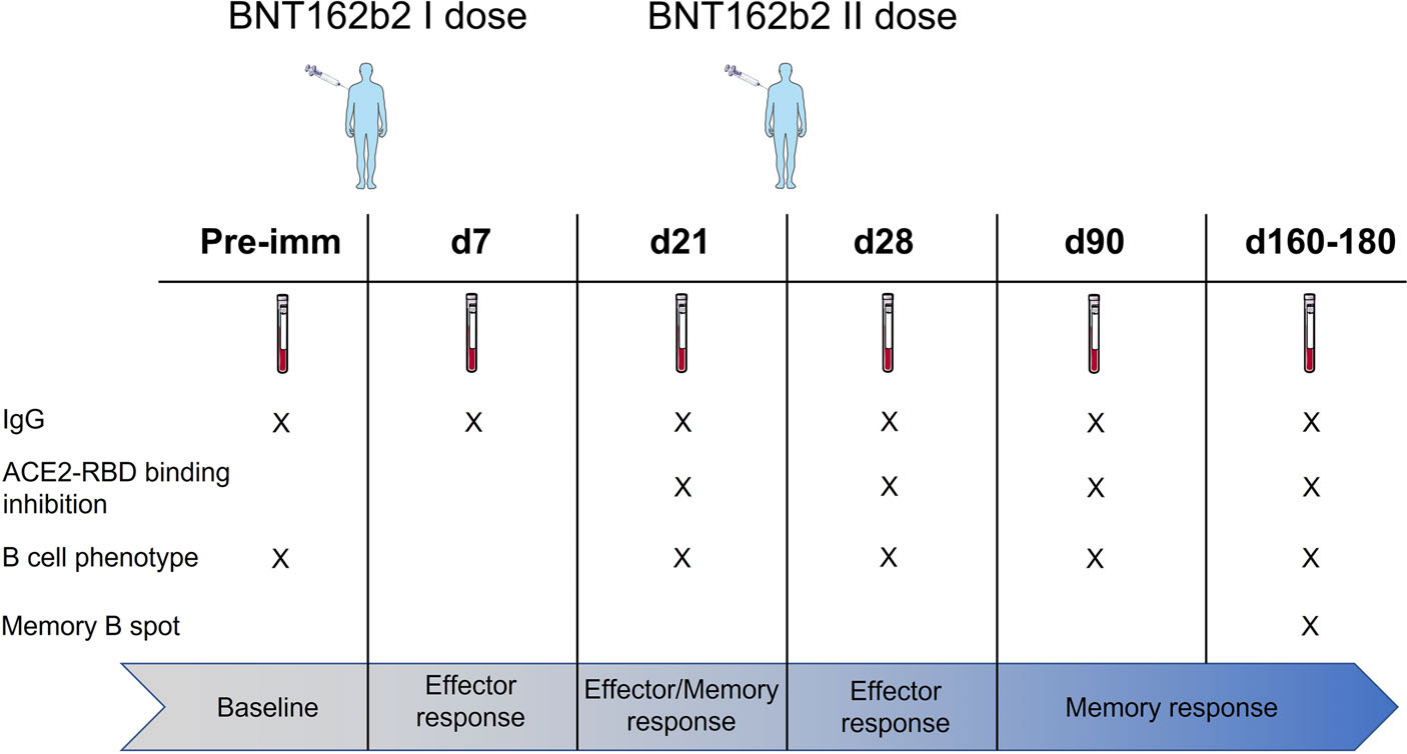
Study design. HCWs (145 subjects) vaccinated with two doses of the BNT162b2 mRNA (Pfizer-BioNTech; Comirnaty) vaccine 3 weeks apart were enrolled in the study. Blood samples were collected at the baseline (day 0), 7, 21, 28 (7 days post second dose), 90 (3 months after the first vaccination dose), and 160–180 (5–6 months after the first vaccination dose) days after vaccination. Plasma and peripheral blood mononuclear cells (PBMCs) were assessed for spike-specific antibodies and memory B cells, respectively.

In this study, we profiled the spike-specific memory B and antibody responses following the administration of the BNT162b2 SARS-CoV-2 mRNA vaccine in Italian HCWs without a laboratory-confirmed history of the SARS-CoV-2 infection. A total of 145 subjects were enrolled in the study, of whom 47 (32.4%) were male with a median age of 47 ± 17 and 98 (67.6%) were female with a median age of 45.1 ± 12.8. The complete characteristics of the volunteers are summarized in **Table S1**. According to the schedule followed for the BNT162b2 mRNA vaccine administration, subjects were vaccinated with two doses, 3 weeks apart. Blood samples were collected at the baseline (day 0), at days 7, 21 (pre-boost), 28 (7 days post-boost), 90 (3 months after the first vaccination dose), and 160–180 (5–6 months after the first vaccination dose) and tested for spike-specific antibodies and memory B cells (**Figure 1**).

### Spike-Specific Antibody Titers and ACE2-RBD Binding Inhibition Activity

**Figure 2:**
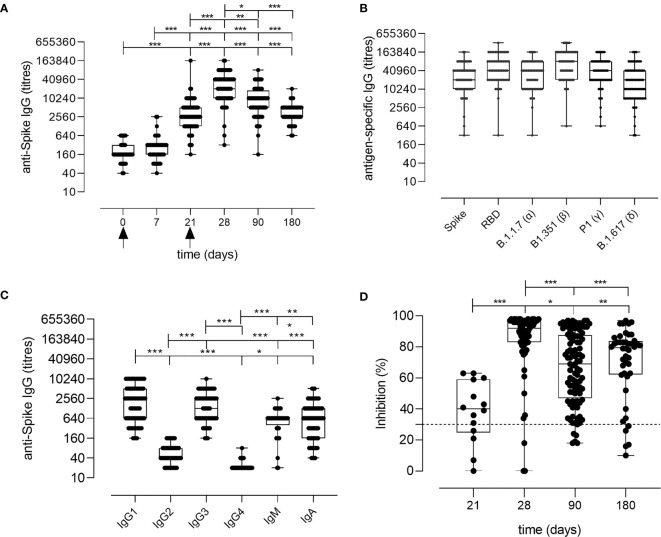
Spike-specific antibody response following BNT162b2 mRNA vaccination. **(A)** Spike-specific IgG analyzed by ELISA in plasma collected 0, 7, 21, 28, 90, 160–180 days after the first dose of the BNT162b2 mRNA vaccine (arrows). Antibody titers are expressed as the reciprocal of the dilution of sample reporting an OD value double respect to the background. Data are shown as box and whiskers diagram showing the minimum and maximum of all the data. **(B)** Titers of IgG anti wild type Spike and RBD, or anti mutated Spike protein (Alpha B.1.1.7, Beta B.1.351, Gamma P.1 and Delta B1.617) in plasma collected at day 28 (7 days after the second vaccination dose). **(C)** Spike-specific IgG1, IgG2, IgG3, IgG4, IgM, and IgA titers in plasma collected at day 28 (7 days after the second vaccination dose). **(D)** Surrogate virus neutralization test performed at days 21, 28, 90, and 160–180 after the first vaccination dose of the BNT162b2 mRNA vaccine. Data are reported as ACE2/RBD binding inhibition percentage with box and whiskers diagram showing the minimum and maximum of all the data. A threshold (dotted red line) was placed at 30% inhibition percentage to discriminate between positive and negative samples. Kruskal-Wallis test, followed by Dunn’s post-test for multiple comparisons, was used for assessing statistical differences between groups. *P ≤ 0.05; **P ≤ 0.01; ***P ≤ 0.001.

Spike-specific IgG levels were assessed at all the time points in the plasma of vaccinated subjects. As shown in **Figure 2A**, a significant increase of spike-specific IgG levels was observed already after the first dose (21 days), with a geometric mean titer (GMT) of 2,869 [95% confidence interval (CI) 2,178 to 3,778; titers range 160**–**163,840; P ≤ 0.001 *vs*. baseline and day 7]. Antibodies peaked 7 days after the second dose with a GMT of 22,120 (95% CI 16,319 to 29,983; range 320**–**163,840; P ≤ 0.001 *vs*. baseline). Significant levels were observed also at day 90 (GMT value of 7,712; 95% CI 6,134 to 9,695; range 160**–**81,920; P ≤ 0.001 *vs*. baseline) and at days 160**–**180 (GMT value of 3,260; 95% CI 2,609 to 4,073; range 640**–**20,480; P ≤ 0.001 *vs*. baseline), despite a progressive significant decline overtime (P ≤ 0.05 between days 28 and 90, P ≤ 0.001 between days 28 and 160**–**180; **Figure 2A**). No statistically significant difference was observed between days 90 and 160**–**180. Binding of plasma IgG to the receptor-binding domain (RBD) of the spike protein was also evaluated at day 28 (GMT value of 50,791; 95% CI 39,232 to 65,754) and no significant difference was observed in respect to the response assessed against the full spike protein (**Figure 2B**).

The analysis of the spike-specific IgG subclasses at day 28 showed a prevalence of IgG1 (GMT of 1,780; 95% CI 1,280 to 2,475; range 160**–**10,240), and IgG3 (GMT of 1,402; 95% CI 1,066 to 1,844; range 160**–**10,240), and a complete absence of IgG2 and IgG4 (GMT of 50 and 22, respectively. **Figure 2C**). Low levels of IgA and IgM were detected, with GMT values of 709 (95% CI 521 to 963; range of 40**–**5,120) and 485 (95% CI 346 to 678; range 20**–**2,560), respectively (**Figure 2C**).

The age of the vaccinated individuals did not have a significant impact on the antibody response, even though a higher GMT of 50,018 (range 5,120**–**163,840) was observed at day 28 in younger individuals (24**–**30 years) compared to subjects older than 31 years (GMT 33,632; range 320**–**163,840; data not shown), in line with other recently reported data (13, 14).

Different SARS-CoV-2 variants, harboring specific mutations in their spike protein, have recently emerged. Antibody binding to Alpha (B.1.1.7 firstly isolated in United Kingdom), Beta (B.1.351, firstly isolated in South Africa), Gamma (P.1, firstly isolated in Brazil), and Delta (B.1.617.2 firstly isolated in India) variants was tested in the plasma collected at day 28 post vaccination to verify if the antibodies generated by the mRNA vaccine were able to bind the mutated spike protein. As shown in **Figure 2B**, antibodies efficiently bound all the variants of the spike protein.

To assess whether the antibodies elicited by BNT162b2 mRNA vaccination were capable of blocking the ACE2/RBD interaction, thus inhibiting the main entrance way of SARS-CoV-2 into human cells, plasma samples collected at days 21, 28, 90, and 160**–**180 after vaccination were tested using a surrogate virus neutralization assay (sVN) (15). As shown in **Figure 2D**, the ACE2/RBD inhibition percentage significantly increased after the second dose (40.73% ± 14.37% at pre-boost versus 85.48% ± 20.50% at day 28) as well as the percentage of subjects with a positive binding inhibition value (from 82% after the first dose to 95% after the second dose). From day 28, a decrease of the inhibition percentage value was observed. A positive correlation according to Pearson test was observed between spike-specific IgG titers and ACE2/RBD inhibition percentage (r = 0.6706; P ≤ 0.001; **Figure S1**).

### Spike-Specific Memory B Cells Generation and Persistence

**Figure 3:**
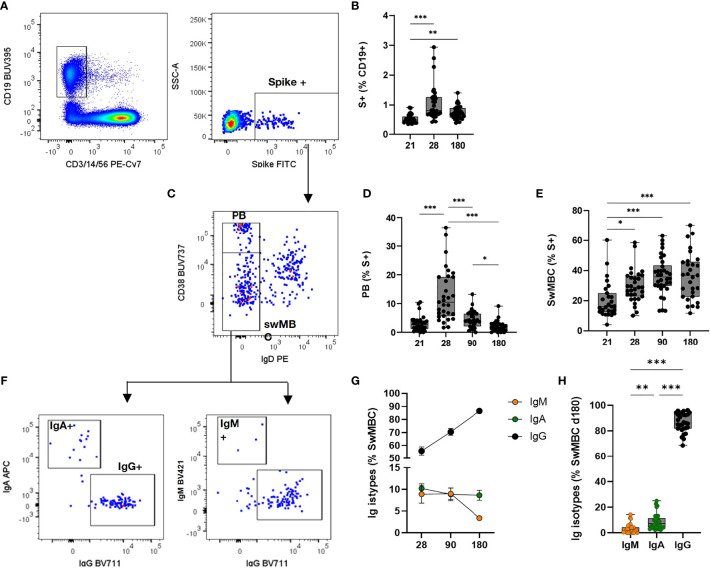
Spike-specific memory B cell response following BNT162b2 mRNA vaccination. Identification of spike-specific B cells by flow cytometry within PBMCs collected at different time points following vaccine administration. **(A)** Gating strategy for identifying CD19^+^ spike-specific B cells (named S^+^ B cells) by multiparametric flow cytometry. **(B)** Percentages of S^+^ B cells in each subjects assessed immediately before (day 21) and after (days 28, 90, and 160–180) the second dose. **(C)** Dot plot analysis of CD38 *vs*. IgD within S^+^ B cells, for identifying IgD^-^CD38 ^high^ plasmablasts (PB) and Ig-switched memory B cells (swMBC). **(D, E)** Percentages of PB **(D)** and swMBC **(E)** in single subjects assessed at days 21, 28, 90, and 160–180. **(F)** Dot plot analysis of IgA, IgG, and IgM expression within swMBC. **(G)**. Mean value (± SEM) of IgM, IgA and IgG swMBC percentages at 28, 90 and 180 days after vaccination. **(H)** Percentages of IgM, IgA, and IgG swMBC in single subjects assessed at days 160–180. Dot plots in **(A, C, F)** are representative from a single subject; values in **(B, D, E, G, H)** are reported as box and whiskers diagram showing the minimum and maximum of all of the data; percentages are reported respect to the parent population (in brackets). Statistical difference was assessed by Kruskal-Wallis test; *P ≤ 0.05; **P ≤ 0.01; ***P ≤ 0.001.

The generation of the B cell response upon vaccination is characterized by the induction of subsets of cells with different functionalities and phenotypes. Here, we profiled the spike-specific B cell response after the administration of two doses of the BNT162b2 mRNA vaccine, and we followed the persistence of memory B cells up to 160**–**180 days after vaccination. B cell responses to vaccination are generally constituted by an early phase of effector response, with plasmablasts (PB)/plasmacells (PC) production, followed by a slower phase of MBC generation. SARS-CoV-2 specific B cells were identified among CD19^+^ cells using a fluorescent spike antigen, and the different B cell subsets were characterized assessing the expression of IgD, CD27, CD38, IgG, IgM, IgA, CD24, CXCR5, and CD11c molecules. Spike-specific cells, assessed before (day 21) and after (days 28, 90, and 160**–**180) the second dose, were detected within the CD19^+^ cells (hereafter named S^+^ B cells, **Figure 3A**). The manual analysis was then compared to the computational one, that allows to profile the B cells in an unbiased way, identifying different cell clusters based on the simultaneous expression of all the surface markers analyzed.

S^+^ B cells significantly increased after the second vaccine dose (P < 0.01 *vs*. pre-boost) and maintained a significantly higher number 6 months after vaccination (P < 0.01 *vs*. pre-boost, **Figure 3B**). The phenotype of the S^+^ B cells changed during the analysis at different time points, in accordance with their effector function. Indeed, the highest frequency of Ig-switched (IgD^-^) CD38^bright^ S^+^ B cells (**Figure 3C**), indicative of PB (16–18), was detected 7 days after the second vaccine dose (**Figure 3D**). These antigen-specific PB significantly increased compared to the pre-boost (about 13% of S^+^ B cells, *vs*. 3% at day 21) and declined overtime (4.3% and 2.5% at 90 and 160**–**180 days, respectively). On the contrary, the switched-memory B cells (swMBC), identified as IgD^-^ CD38^int/low^ (**Figure 3C**), increased after the second dose, starting with a percentage of 16% at day 21 up to 36% at days 90 and 160**–**180 (**Figure 3E**). Memory S^+^ B cells analyzed after the booster dose included IgM^+^, IgA^+^, or IgG^+^ cells and their frequency changed according to the time elapsed since the last dose (**Figure 3G**). IgG^+^ cells significantly increased reaching a frequency of 86 ± 7% from 55 ± 17% at day 28, while IgA^+^ were stably maintained (from 10 ± 6% to 8.9 ± 6.3%) and IgM^+^ cells declined overtime (from 8.8 ± 10% to 3.2 ± 3% at 7 days after boosting and month 6, respectively) (**Figure 3H**).

### Identification of Spike-Specific B Cell Clusters by Automated Analysis

The computational analysis of multiparametric flow cytometry data is a powerful tool for dissecting all the possible cell phenotypes present in a sample, in an operator-independent unbiased way (19). To automatically profile the spike-specific B cells elicited by the mRNA vaccine and determine their modulation at the different time points following vaccine administration, the S^+^ B cells were analyzed employing the FlowSOM clustering algorithm (20). This approach considers the distribution of all surface markers simultaneously, allowing to characterize most of the possible phenotypes in an unbiased way, including the unexpected ones (11). Groups of cells with similar markers expression are automatically grouped into metaclusters (M). FlowSOM uses an algorithm based on a self-organizing map (SOM) built assigning similar cells to nodes (20). In a second optional step, similar nodes are metaclustered together with a hierarchical process (21). The metaclustering step facilitates the interpretation of the results, as the high number of nodes in the SOM cannot be easily associated with different cell populations.

**Figure 4:**
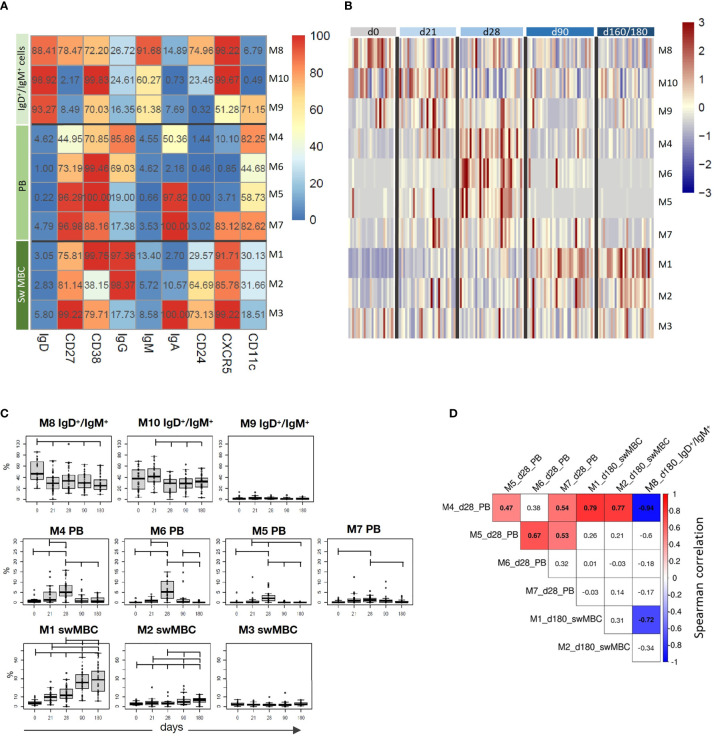
Computational analysis of spike-specific B cells following BNT162b2 mRNA vaccination. **(A)** Heatmap of metaclusters from the FlowSOM analysis of CD19^+^Spike^+^ B cells. Each marker is reported in column, while the different metaclusters (M) are reported in rows. The percentage of cells positive for each marker is reported inside the heatmap boxes, and visualized with a color scale from blue (0%) to red (100%). **(B)** Heatmap reporting the normalized frequency of each metacluster (rows) assessed in each subject (columns) in a color scale from blue (low) to red (high). Columns were grouped by the sampling day, as reported above the heatmap. **(C)** Box and whiskers plots showing the frequency of each metacluster at different time points. Values from individual samples were reported as circles. Mann–Whitney test corrected for multiple tests (Benjamini-Hochberg method) was used for assessing the statistical differences between different time points; statistical significance was defined as FDR < 10^-2^. **(D)** Correlation analysis between significantly modulated metaclusters at day 28 (M4, M5, M6, and M7) and at day 180 (M1, M2, and M8). Multiple correlations were visualized as matrix with the Spearman’s correlation coefficient values reported. Significant values FDR < 0.05; correlation coefficient between 1 [red] and –1 [blue]. Only significant correlation were colored.

The computational analysis of the surface markers on spike-specific B cells was performed on all vaccinated subjects at all time-points simultaneously. The algorithm identified 10 metaclusters grouping different B cell populations, such as the double positive IgD^+^/IgM^+^ cells, PB, and isotype-switched MBC subsets (**Figure 4A**). Different FlowSOM metaclusters of spike-specific B cells are visualized in the heatmap in which each metacluster is reported in row and surface markers in column (**Figure 4A**) The percentages of positive cells for each marker inside the metacluster is visualized as a color scale from blue (0% of positive cells) to red (100%). The modulation of the different metaclusters at baseline (day 0), before the boosting (day 21), and after the second dose (days 28, 90, and 160**–**180) is reported in each subject in the heatmap in **Figure 4B**. This analysis allows to visualize the frequency of each metacluster in each subject at different time points following vaccination and to identify the trend of the various B cell subsets during the different phases of the B cell response.

While spike-specific IgM/IgD double positive subsets (M8, M9, and M10) were mostly observed at the baseline or after the first vaccine dose (day 21), Ig-switched cells (IgD^-^CD27^+^; M4, M5, M6, M1, M2, M7, M3) significantly increased after the second vaccine dose (**Figure 4B**). In particular, 7 days after the vaccine administration (d28), M4, M5, and M6 representing antigen-specific PB (CD27^+^, CD38^+^ CD24^-^ CXCR5^-^ CD11c^+^) (22) were strongly expressed by most of the subjects, and declined at later time points. Some of these PB were IgA^+^ (M5 and M7) and others IgG^+^ (M4 and M6). Three and six months after vaccination, the phenotype of most of S^+^ B cells changed into memory cells, as clearly shown by the downregulation of CD11c and the increased expression of both CXCR5 and CD24 molecules. Even though some of these memory cells were still IgA^+^ (M3), the majority switched to IgG (M1 and M2). The frequencies of metaclusters are shown in the heatmap in **Figure 4A** and the statistical analysis of their modulation at the different time points is reported in **Figure 4C**. This analysis was obtained extracting the frequencies of the identified metaclusters from the FCS files of each subject at each time points. As visualized in **Figure 4B**, PB were significantly increased early after boosting (M4, M5, M6, and M7), while IgG-switched MBC (M1 and M2) significantly increased at days 90 and 160**–**180 (**Figure 4C**).

As clearly shown in **Figure 4B**, we can observe a time-dependent modulation of the B cell response, that moving from CD27^+^ IgD^+^ IgM^+^ CXCR5^+^ cells at early time points (M8, M10 and M9), differentiate into both IgG or IgA effector cells (CD27^+^ IgD^-^ CD38^+^ CXCR5^-^ CD11c^+^) at 7 days after the second dose (M4, M6, M5 and M7), and then develop into memory cells (CD27^+^ IgD^-^ CD38^+^ CXCR5^+^ CD24^+^ CD11c^-^, M1 and M3), perfectly correlating with the expected B cell response kinetic.

The multiple correlation analysis, performed to assess the relationship between the most significant metaclusters at days 28 and 160**–**180, showed that the frequency of different PB (M4, M5, M6, and M7) significantly correlated each other at day 28, and that M4 positively correlated with the frequency of IgG^+^ switched memory cells (M1 and M2) at day 180, and inversely correlated with the most undifferentiated B cells (M8) at day 180 (**Figure 4D**).

The automated analysis not only corroborates what was observed with the manual gating strategy, but consistently improves the characterization of the B cell subsets elicited by the SARS-CoV-2 vaccination.

### Antibody Secretion by Reactivated Memory B Cells

**Figure 5:**
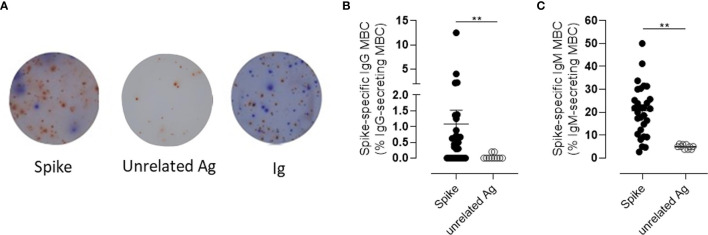
Spike-specific memory B cell response following BNT162b2 mRNA vaccination evaluated by B-cell ELISPOT. **(A)** Representative images of ELISpot wells coated with spike (left), an unrelated antigen (center), or anti-immunoglobulin (Ig, right) and developed in blue and red for IgG and IgM, respectively, after incubation of PBMCs. Cells were collected 160–180 days following the first dose of mRNA BTN162b2 vaccination, and restimulated *in vitro* with B-Poly-S for 4 days to induce resting MBC differentiation into antibody-secreting cells. **(B, C)** The frequencies of spike-specific MBCs secreting IgG **(B)** or IgM **(C)** antibodies are reported as percentages of total MBCs producing antibodies of the respective isotype. Bars indicate mean ± SEM. Mann–Whitney test, followed by Dunn’s post-test for multiple comparisons, was used for assessing the statistical difference between Spike-specific and unrelated antigen-specific B cells. **P ≤ 0.01.

To assess the functionality of the circulating S^+^B cells present in the blood of vaccinated subjects 6 months after vaccination, the frequency of spike-specific antibody-secreting cells was determined in PBMCs by the ELISpot assay (**Figure 5A**). As control, PBMCs from the same subjects were restimulated with an unrelated antigen Spike-specific IgG-secreting cells were found in the peripheral blood of 66% of the subjects tested, with a mean frequency of about 1% of the total IgG-secreting cells (**Figure 5B**). Circulating spike-specific IgM-secreting B cells were found in 100% of the subjects, with a percentage of about 20% of the total IgM-secreting cells (**Figure 5C**). As shown with the automated analysis, IgM^+^ memory cells were also positive for IgD. These subsets of lymphocytes were excluded from the cells reported in **Figure 3G**, in which only Ig-switched memory B cells were characterized.

Taken together, these data profile the kinetic of the spike-specific B response elicited by the BNT162b2 mRNA vaccine, highlighting the slow decline of spike-specific antibody levels overtime that is accompanied by the induction of circulating spike-specific IgM and IgG switched memory B cells, that persist 6 months after the mRNA BTN162b2 vaccination.

## Discussion

In this work, we demonstrate that spike-specific memory B cells, capable of reactivation following antigen encounter, persist in the blood of vaccinated subjects 6 months after the administration of the BNT162b2 SARS-CoV-2 mRNA vaccine. Concomitant to antibody reduction, spike-specific memory B cells, mostly IgG class-switched, increase in the blood of vaccinees and persist 6 months after vaccination. Considering the natural decline of spike-specific circulating antibodies, our results highlight the importance of profiling the antigen-specific memory B cell response, a crucial biomarker of vaccine immunity that could be particularly important to monitor vaccine responsiveness and long-term memory persistence.

While most available data of the BNT162b2 vaccine are on the antibodies elicited upon the first vaccine dose in healthy or SARS-CoV-2 previously infected subjects or at early time points following the second vaccine dose (7, 23–28), our study profiles the spike-specific antibody response and memory B cells up to 6 months after vaccination, contributing to better understand the BNT162b2 vaccine immunogenicity in SARS-CoV-2 naive subjects.

Through a computational analysis of flow cytometry data, we profiled the spike-specific B cell response, identifying spike-specific PB 7 days after the second vaccine dose, and Ig-switched memory B cells that increased at month 3 and still persisted at month 6 post vaccination (**Figures 3D, E**). The transient appearance of PB in blood with a peak at 7 days after the BNT162b2 mRNA vaccine administration is in line with what was observed with other vaccines, such as attenuated yellow fever strain YF-17D, inactivated influenza vaccine, and tetanus vaccine (22).

Most of the spike-specific memory B cells were IgG^+^, but also IgD/IgM double positive cells were detected (**Figure 4A**). The frequency of spike-specific IgG^+^ plasmablasts present 7 days after the second vaccine dose positively correlated with the frequency of IgG^+^ memory B cells at day 180, suggesting a predictive value of PB frequency for spike-specific memory B cells generation (**Figure 4D**). Systems biology approaches aimed to identify immunological parameters predictive of long-term responses elicited by vaccination against influenza have also identified the early induction of PB as a potential biomarker of memory B cells generation (29).

In the context of viral infections, it is critical to analyze the persistence of the antibody response and to measure protective antibody titers by functional assays, as well as to assess the presence of circulating spike-specific memory B cells that can be reactivated following an antigen encounter. Rapid activation of memory B cells and their differentiation into antibody-secreting PBs is essential for providing antibodies capable of neutralizing the virus (22). Our data show that upon *in vitro* restimulation, circulating memory spike-specific B cells elicited by the BNT162b2 vaccine were capable of reactivation and differentiation into IgG-secreting cells (in 66% of vaccinated subjects) or IgM-secreting cells (100% of vaccinated subjects; **Figures 5B, C**).

Parallel to the dissection of the B cellular response, we monitored the antibody responses against the spike protein for up to 6 months after vaccination, observing a peak of IgG 7 days after boost and the persistence of significant levels up to 6 months, despite a progressive decline overtime.

Nevertheless, IgG in the plasma collected 6 months after vaccination still inhibited the *in vitro* binding between RBD and the ACE2 receptor, in all the samples assessed. This surrogate virus neutralization assay has been demonstrated to concord with the gold standard 90% plaque reduction neutralization tests (PRNT_90_) for SARS-CoV-2 antibody detection in human sera (30). At the peak of response to the second vaccine dose, all subjects showed responses to all tested variants (Alpha, Beta, Gamma, and Delta), as recently reported by Pegu et al. who demonstrated the persistence of binding and functional antibodies against variants in most mRNA vaccinated subjects for 6 months after the second vaccine dose (31).

IgG1 and IgG3 were the most abundant IgG subclasses produced, but also spike-specific IgA were released according to the detection of IgA^+^ PB and memory cells at days 28 and 180, respectively. A similar distribution of IgG subclasses has been previously observed in SARS-CoV-2 infected patients and has been negatively associated with the viral load in nasopharyngeal swab (32).

To summarize, 6 months after the vaccination of the subjects without a previous history of SARS-CoV-2 infection with two doses of the BNT162b2 mRNA vaccine, concomitant to antibody reduction is observed a consistent and persistent spike specific IgG-memory B cell response, with cells capable of reactivation following antigen encounter. Spike specific IgG antibodies are still present in the blood, with a demonstrated ACE2/RBD binding inhibition activity, even though the trend of the antibody curve shows a physiological decline overtime (**Figures 2A–D**). The induction and the longevity of circulating spike-specific memory B cells, capable of reactivating into a novel wave of plasmablasts and plasma cells producing spike-specific antibodies following a secondary antigen encounter, is the critical biomarker indicating the capacity of an effective response to pathogen encounter.

These data contribute to provide an answer to the open question on the duration of the memory response to the BNT162b2 vaccine and on the possible need and timing of repeated booster doses of a COVID-19 vaccine in healthy subjects (33). This type of analysis could be particularly relevant when applied to fragile patients that, due to the immune impairment associated with their primary disease (34, 35) and/or age (36–38), are particularly at a high risk for severe disease and death related to COVID-19. In these subjects, the immune response elicited by vaccination can be affected by disease, age and treatment, therefore, the possibility of investigating not only the antibody response but characterizing also the behavior of their memory B cells compartment can be instrumental for defying the vaccination policy most adequate for each specific patient category.

In conclusion, these data demonstrate that the mRNA BNT162b2 vaccine elicits robust B cell immunity 6 months after vaccination, with persistent spike-specific memory B cells crucial for a rapid response to SARS-CoV-2 virus encounter, and are particularly important to guide vaccination schedules and policies.

## Data Availability Statement

The original contributions presented in the study are included in the article/**Supplementary Material**. Further inquiries can be directed to the corresponding authors.

## Ethics Statement

The studies involving human participants were reviewed and approved by the local Ethical Committee for Clinical experimentation of Regione Toscana Area Vasta Sud Est (CEASVE), protocol code 18869 IMMUNO_COV v1.0 of November 18, 2020, approved at December 21, 2020. The patients/participants provided their written informed consent to participate in this study.

## Author Contributions

AC, DM, GP, MF, and FM conceived the study. IR, MM, BR, MF, GP, and FM enrolled the patients. GP, FF, JP, SA, and AC processed the samples. MD, GP, SA, and AC managed the database. AC, GP, FF, JP, and EP carried out the immunological analysis. AC, FF, SL, GP, and DM analyzed the data. AC, DM, and GP wrote the manuscript. AC, DM, FM, and GP supervised the study. DM provided the financial support. All authors contributed to the article and approved the submitted version.

## Conflict of Interest

The authors declare that the research was conducted in the absence of any commercial or financial relationships that could be construed as a potential conflict of interest.

## Publisher’s Note

All claims expressed in this article are solely those of the authors and do not necessarily represent those of their affiliated organizations, or those of the publisher, the editors and the reviewers. Any product that may be evaluated in this article, or claim that may be made by its manufacturer, is not guaranteed or endorsed by the publisher.
