# Supplementary material for: Evidence of SARS-CoV-2-Specific Memory B Cells Six Months After Vaccination With the BNT162b2 mRNA Vaccine
Source: Front Immunol. 2021 Sep 28;12:740708. doi: 10.3389/fimmu.2021.740708 (PMC8505800; doi:10.3389/fimmu.2021.740708)

Table S1. Characteristics of the study cohort

	TOTAL	PRE-VACCINE	POST-VACCINE DOSE 1	POST-VACCINE DOSE 2
N	145	63 (43.5%)	80 (55.2%)	102 (70.3%)
Age In Years, Mean (SD)	48.8 (14.3)	47.7 (14.9)	41.7 (12.5)	44.2 (13.7)
Sex, N				
Female	98	37	55	72
Male	47	26	25	30

Figure S1. Correlation between IgG ELISA titres and surrogate virus neutralization. Antibody titres are expressed as the reciprocal of the dilution of sample reporting an OD value double respect to the background. The surrogate virus neutralization was expressed as ACE2/RBD inhibition percentage. Pearson correlation test was used to assess the correlation between the two data groups.

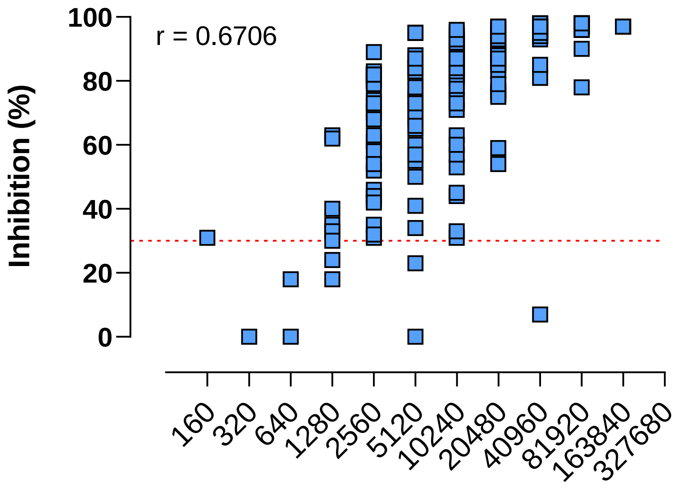

Supplement: Supplementary file 1 [file DataSheet_1.pdf]
